# FedDyH: A Multi-Policy with GA Optimization Framework for Dynamic Heterogeneous Federated Learning

**DOI:** 10.3390/biomimetics10030185

**Published:** 2025-03-17

**Authors:** Xuhua Zhao, Yongming Zheng, Jiaxiang Wan, Yehong Li, Donglin Zhu, Zhenyu Xu, Huijuan Lu

**Affiliations:** 1 School of Electronic Information, Zhejiang Guangsha Vocational and Technical University of Construction, Dongyang 322103, China; 2 School of Software, East China University of Technology, Nanchang 330013, China; ymzheng@ecut.edu.cn; 3 Jiangxi Provincial Key Laboratory of Multidimensional Intelligent Perception and Control, Ganzhou 341000, China6720230827@mail.jxust.edu.cn (Y.L.); 4 School of Computer Science and Technology, Zhejiang Normal University, Jinhua 321004, China; 5 Faculty of Information Science and Engineering, Ocean University of China, Qingdao 266100, China; 6 College of Information Engineering, China Jiliang University, Hangzhou 310018, China

**Keywords:** federated learning, knowledge distillation, genetic algorithm, catastrophic forgetting

## Abstract

Federated learning (FL) is a distributed learning technique that ensures data privacy and has shown significant potential in cross-institutional image analysis. However, existing methods struggle with the inherent dynamic heterogeneity of real-world data, such as changes in cellular differentiation during disease progression or feature distribution shifts due to different imaging devices. This dynamic heterogeneity can cause catastrophic forgetting, leading to reduced performance in medical predictions across stages. Unlike previous federated learning studies that paid insufficient attention to dynamic heterogeneity, this paper proposes the FedDyH framework to address this challenge. Inspired by the adaptive regulation mechanisms of biological systems, this framework incorporates several core modules to tackle the issues arising from dynamic heterogeneity. First, the framework simulates intercellular information transfer through cross-client knowledge distillation, preserving local features while mitigating knowledge forgetting. Additionally, a dynamic regularization term is designed in which the strength can be adaptively adjusted based on real-world conditions. This mechanism resembles the role of regulatory T cells in the immune system, balancing global model convergence with local specificity adjustments to enhance the robustness of the global model while preventing interference from diverse client features. Finally, the framework introduces a genetic algorithm (GA) to simulate biological evolution, leveraging mechanisms such as gene selection, crossover, and mutation to optimize hyperparameter configurations. This enables the model to adaptively find the optimal hyperparameters in an ever-changing environment, thereby improving both adaptability and performance. Prior to this work, few studies have explored the use of optimization algorithms for hyperparameter tuning in federated learning. Experimental results demonstrate that the FedDyH framework improves accuracy compared to the SOTA baseline FedDecorr by 2.59%, 0.55%, and 5.79% on the MNIST, Fashion-MNIST, and CIFAR-10 benchmark datasets, respectively. This framework effectively addresses data heterogeneity issues in dynamic heterogeneous environments, providing an innovative solution for achieving more stable and accurate distributed federated learning.

## 1. Introduction

As a key distributed model training paradigm, federated learning enables collaborative learning across clients while protecting data privacy [1]. Unlike centralized learning, FL updates the global model by aggregating locally trained models rather than exchanging raw data, making it particularly suitable for privacy-sensitive application scenarios such as healthcare [2], finance [3], and the Internet of Things [4]. However, dynamic and ever-changing client data environments in real-world scenarios make the static data assumption ineffective in addressing the complexity of heterogeneous data distributions and incremental learning tasks.

In such dynamic environments, FL faces numerous challenges: first, data heterogeneity among clients [5] leads to inconsistencies in optimization, which in turn causes bias in the global model and reduces performance; second, as each client continuously receives new data or tasks, the problem of catastrophic forgetting [6] becomes particularly prominent, as the model struggles to retain knowledge of previous tasks while adapting to new ones; finally, limited communication bandwidth and computational resources [7] in federated learning environments further exacerbate these problems.

Although a large amount of research has focused on mitigating data heterogeneity and improving communication efficiency, most existing methods are based on the static data assumption, which exhibits significant limitations when faced with dynamic scenarios. For example, techniques based on proximity regularization [8], clustering aggregation [9], and contrastive learning [10] have shown some potential in reducing client drift, but are difficult to apply in environments where data distributions and tasks are constantly evolving.

In dynamic and heterogeneous scenarios where each client’s data evolve over time and exhibit significant differences, traditional federated learning frameworks typically assume that data distributions are static and consistent. This assumption encounters challenges when seeking to meet the needs of dynamically evolving client tasks in real-world applications, resulting in limited performance of the global model when adapted to diverse distributions and new tasks. In addition, the distribution heterogeneity among clients further amplifies the optimization inconsistency during model training, making it difficult to guarantee the generalization ability and stability of the global model.

To address these issues, we draw inspiration from the adaptive regulatory mechanisms in biological systems and propose a federated learning framework, which we call FedDyH, to tackle the dynamic heterogeneity of client data in evolving heterogeneous scenarios. First, a cosine classifier is applied to the FedDyH framework to address the heterogeneity and imbalance of client data in dynamic heterogeneous federated learning, similar to how immune systems maintain population-level defense strategies through HLA polymorphism. Next, inspired by the concept of knowledge distillation, the global model is used to transfer shared knowledge to the clients. This process is analogous to the transmission of information between cells via gap junctions. Additionally, we incorporate regularization strategies to mitigate the catastrophic forgetting problem caused by dynamic changes in the data. To optimize the objective function, we introduce a genetic algorithm that simulates the biological evolution process. Mechanisms such as gene selection, crossover, and mutation are used to optimize the hyperparameter configuration, allowing the model to adaptively find the best hyperparameters for distillation loss and orthogonality loss in a constantly changing environment. With this series of strategies, FedDyH effectively addresses data changes in dynamic environments, improving both the global model’s performance and the adaptability of clients. This opens up new possibilities for practical applications of federated learning.

The main contributions of this paper are summarized as follows:

- We propose a novel federated learning framework called FedDyH, which overcomes the performance degradation issues of existing methods such as FedAvg and FedProx under the static heterogeneity assumption. FedDyH specifically focuses on addressing the dynamic heterogeneity of client data in real-world scenarios.
- We incorporate a regularization mechanism combining orthogonality constraints and knowledge distillation during the training process to ensure the stability and effectiveness of the model in dynamic data environments.
- By introducing a genetic algorithm (GA), we dynamically optimize the weights of the distillation loss and orthogonality constraint loss. Compared to traditional methods that rely on fixed hyperparameters or manual tuning, FedDyH enables the model to adapt to the requirements of different training stages, thereby improving both convergence speed and accuracy.
- We conducted extensive experiments on the MNIST, Fashion-MNIST, and CIFAR-10 datasets to evaluate the performance of the FedDyH framework under varying client selection rates and degrees of heterogeneity. Our experimental results show that FedDyH significantly outperforms other federated learning (FL) baseline methods.

The remainder of this paper is organized as follows: Section 2 provides a brief summary of related work; Section 3 details the principles and implementation process of the innovative techniques applied in FedDyH; Section 4 verifies the effectiveness and performance of FedDyH through experiments while discussing the experimental setup, dataset selection, implementation details, and other factors that may have influenced the results; finally, Section 5 concludes the paper with a summary of the main content.

## 2. Related Work

### 2.1. Federated Learning with Heterogeneous Data

The FedAvg [11] benchmark framework for federated learning iteratively updates the global model by adopting a periodic weighted averaging strategy to aggregate the local model parameters of participating clients. However, when facing data scenarios without an independently identical distribution (non-IID), the algorithm causes inconsistency in the direction of gradient update due to deviation of the client data distribution from the global distribution. In turn, this triggers critical problems such as decreased model convergence speed and degradation of generalization performance. To overcome this theoretical bottleneck, researchers have proposed various improvements from multiple dimensions, including but not limited to dynamic client selection mechanisms, adaptive model aggregation strategies, and personalized federated learning frameworks based on meta-learning. These methods primarily focus on enhancing both the local training process at the client side and the global aggregation process at the server side.

In terms of local training on the client side, many studies have focused on mitigating the impact of data heterogeneity by incorporating regularization techniques during the local training process. For example, FedProx [12] adds a proximal term to the local objective function, limiting the deviation of the local model update from the global model. This ensures that local training is constrained by global information to some extent, thereby reducing client drift caused by differences in data distribution. Moon [13] adopts a model comparison regularization method which compares the feature representations of the global model, the current local model, and the previous round’s local model. This approach encourages the local model to align its feature space with that of the global model, enhancing the consistency of the model across different data distributions. FedAlign [14] improves model generalization and robustness by introducing Lipschitz continuity regularization in the last layer of the network. However, all of these methods have two main shortcomings: first, while they limit drift, they also constrain local convergence, resulting in relatively less new information being collected in each communication round; second, they incur significant memory and computational overhead.

In addition, many studies have explored different weighting and normalization methods to improve global model aggregation during the global aggregation process on the server side. FedNova [15] normalizes the client update weights based on local computation resources to ensure that clients with different computational capabilities and data scales contribute reasonable information, thereby preventing individual clients from dominating the global model update direction. However, this method relies on accurate local computation information. In practical applications, accurately measuring and synchronizing computation resources may be challenging. Inaccurate computation information can affect the normalization of the weights, degrading the performance of the global model. A number of methods weight their client updates based on the amount of data, assigning more weight to clients with larger datasets during aggregation. While this helps to make full use of information from large-scale data, it may overlook differences in data quality. If a client provides a large dataset with poor data quality, this could introduce erroneous information into the global model. FedDF [16] utilizes an unlabeled external dataset on the server to distill the global model’s knowledge into the client models, which promotes knowledge transfer by matching outputs. However, obtaining a suitable external dataset can be challenging, and hyperparameter tuning during the knowledge distillation process is complex. If the hyperparameters are not set properly, it may hinder effective knowledge transfer or even mislead the model during training.

To highlight the advantages of FedDyH over traditional federated learning methods, we compared it with several commonly used approaches, including FedAvg, FedProx, SCAFFOLD, FedSAM, and FedDercorr. In Table 1, we provide a detailed comparison of the various methods across key metrics, including adaptability to dynamic heterogeneous scenarios, optimization strategies, catastrophic forgetting mitigation, hyperparameter optimization, data heterogeneity handling, and model generalization capability. The comparison demonstrates that FedDyH outperforms other methods in multiple aspects, particularly in handling dynamic client data changes, adaptive optimization of objectives, and enhancing the generalization ability of the global model, showcasing its significant advantages.

### 2.2. Knowledge Distillation

Knowledge distillation, originally proposed by Hinton et al., is used to effectively transfer knowledge from complex models to lightweight models, thereby enhancing the learning ability and generalization performance of the model [17]. By learning the predicted probability distribution from the teacher model, the student model can capture deeper information and optimize its learning performance. In federated learning (FL), knowledge distillation has been widely studied in recent years as a key technique for mitigating the data distribution differences among clients. For example, FedDistill [18] employs group distillation, which subdivides categories based on their frequency in the local dataset to facilitate concentrated distillation for underrepresented categories, thereby improving the global model’s generalization ability. Additionally, PKT [19] suggests that knowledge from the teacher model can be transferred through probability distributions by minimizing the difference between the outputs of the teacher and the students. The Logit normalization method [20] optimizes the knowledge distillation process by introducing a preprocessing step based on the Z-score, allowing the student model to better learn the inherent relationships of the teacher model without forcing a match in the range and variance of logits. This effectively improves the performance of the student model. These methods demonstrate the feasibility of using knowledge distillation to address the data heterogeneity problem in federated learning.

### 2.3. Genetic Algorithm

Genetic algorithms are known for their excellent robustness and adaptability, and have found wide use in various optimization problems. They have shown significant advantages, particularly in addressing complex distributed systems and large-scale data processing tasks. Specifically, GAs simulate the natural evolution process by encoding potential solutions as “chromosomes” and iteratively optimizing them through genetic operations such as selection, crossover, and mutation. This process allows GAs to search for approximate optimal solutions in the solution space in a flexible and efficient manner, significantly improving both the efficiency and accuracy of problem-solving. For example, Kang et al. (2023) [21] proposed a GA-based client selection method which effectively reduced communication overhead while maintaining the performance of the global model.

In federated learning, GAs have been applied to optimize model aggregation strategies. Zeng et al. (2024) [22] proposed the FedGR framework, which uses genetic algorithms to group clients and employs a relay strategy for training and aggregating models, significantly improving the accuracy of federated learning. Fu et al. (2024) [23] applied genetic algorithms to optimize their base model by reducing the number of hidden units, which further optimizes the federated learning framework by improving the efficiency of inference time and storage space. Hybrid approaches combining GAs with other optimization techniques have also shown excellent performance in addressing challenges in federated learning. Zhu et al. (2023) [24] proposed a Manta Ray foraging optimization based on a mechanics game and progressive learning. By introducing variable spiral factors and a matching game mechanism akin to the fitness evaluation and selection mechanisms in genetic algorithms, their algorithm improved both global search ability and local accuracy, making it suitable for solving multiple optimization models. Therefore, our work suggests combining GAs with federated learning to enhance the training efficiency and accuracy of the resulting models.

### 2.4. Orthogonality-Related Methods

Orthogonality has been widely recognized as an effective technique for addressing the issues of gradient vanishing and gradient explosion in deep learning, and its advantages have gradually extended to the field of federated learning (FL). In FL scenarios, data heterogeneity and inconsistencies in model optimization pose significant challenges, while orthogonality-based approaches can provide effective solutions to these problems.

Orthogonal matrices satisfy $W^{T}W=WW^{T}=I$, which helps to maintain the stability of norms during the optimization process. This property is utilized in FL to enhance the stability of model updates and mitigate the issue of client drift. For example, Saxe, McClelland, and Ganguli (2014) [25] proposed orthogonal weight initialization. Compared to Gaussian initialization, orthogonal initialization performs better in deep networks, which has inspired its application in federated learning frameworks as well.

In the context of FL, orthogonality is typically introduced as a regularization term. Orthogonal constraints can be imposed by calculating the Frobenius norm difference between the Gram matrix of the weight matrix W and identity matrix I, i.e., $|WW^{T}{-I|}_{F}^{2}$. The gradient of this regularization term is $4W(W^{T}W-I)$. This ensures the stability of the model during training. Brock, Lim, Ritchie, and Weston (2017) [26] further expanded this approach by applying L1-norm orthogonal regularization to GANs. This framework has also been adapted to FL, where it is used to reduce the inconsistency between local models and the global model.

## 3. Method

This chapter mainly introduces the FedDyH framework and related innovative techniques. First, Section 3.1 provides a detailed explanation of the optimization objectives and implementation methods of the framework. In Section 3.2, we discuss the classifiers, including the reasons for their selection and their working principles. Section 3.3 introduces the regularization strategies used for model training, with a focus on their role in the optimization process. Finally, Section 3.4 presents the genetic algorithm and explains its application during optimization.

### 3.1. FedDyH Framework

Unlike traditional static federated learning, the dynamic federated learning approach proposed in this study involves clients being exposed to new datasets after each round of global communication. Additionally, each client randomly receives a different amount of new data after every training round, simulating a real-world application scenario in which the data continuously change and grow. In such a dynamic environment, the optimization objective can be expressed as follows:

(1) $$\begin{array}{c} \min\left\{f\left(\omega \right)=\frac{1}{m}\sum_{i\in \left[m\right]}f_{i}\left(\omega ,D_{i}\left(t\right)\right)\right\} \end{array}$$

(2) $$f_{i}\left(\omega ,D_{i}\left(t\right)\right)≜E_{\varepsilon_{i}∼D_{i}\left(t\right)}\left[f_{i}\left(\omega ,\varepsilon_{i}\right)\right]$$

where f:Rd→R is the global objective function, ω represents the model parameters, *m* is the total number of clients, and [m] denotes the set of all clients; moreover, $D_{i}\left(t\right)$ represents the new dataset of client i in training round t, simulating the continuous growth and change of data. Dataset division is first performed using a Dirichlet distribution, then the data volume is dynamically increased on this basis. Here, $f_{i}\left(\omega ,D_{i}\left(t\right)\right)$ is the loss function for client *i*, while $\varepsilon_{i}$ is a data sample randomly selected from the local data distribution $D_{i}\left(t\right)$ of the client. Due to data heterogeneity, the data samples of each client may differ.

**Figure 1:**
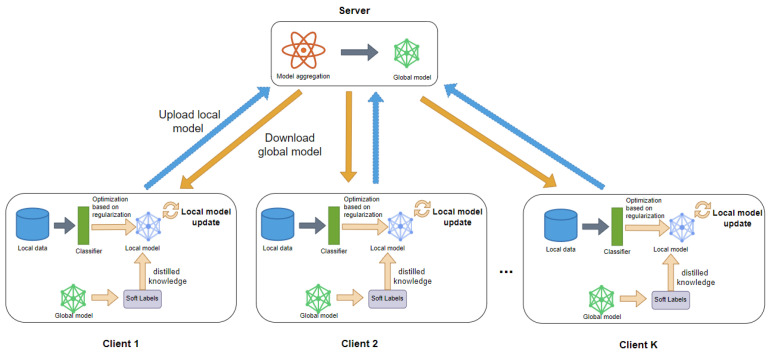
Basic federated learning architecture.

To address the dynamic evolution characteristics of real-world data, the FedDyH framework draws inspiration from the coordinated adaptation mechanisms of biological systems. In FedDyH, multiple clients collaborate with a central server, similar to how multicellular organisms maintain their functionality and evolve as a whole in response to changing environments. As illustrated in Figure 1, by simulating the “cooperation” mechanism among cells, the proposed framework effectively preserves local feature patterns (e.g., staining preferences in pathology images from specific hospitals) while mitigating catastrophic forgetting and enhancing the global model’s ability to adapt to diverse data distributions. In each training round, each client first uses a classifier to categorize and optimize the data by class. Then, during the local training process, the client optimizes the model through a regularization mechanism, which includes using a genetic algorithm to dynamically adjust the weights of the orthogonality constraint loss and knowledge distillation loss within the regularization mechanism. Clients can be viewed as “cells”, with each cell performing local optimization based on its own data before uploading the updated local model to the server. This approach simulates the process of multicellular aggregation and information sharing. The server receives all client-uploaded models and generates a new global model through an aggregation mechanism. After the global model has been formed, it is distributed back to the clients to serve as the foundation for the next training round.

The overall FedDyH algorithm is shown in Algorithm 1. In each round, the server sends the global model to each participant, receives the local models from the participants, and updates the global model using weighted averaging. During local training, each participant uses a classifier to optimize the data categories and combines regularization mechanisms to optimize the local model.
**Algorithm 1** FedDyH  1:**Initialization:** $w_{0}$, $\rho_{0}$, $\Delta^{0}=0$, learning rates $\eta_{l}$, $\eta_{g}$, number of communication rounds r and number of local epochs *K*, Convolution kernels to be orthogonalized $\tilde{W}$.  2:**for** r=0,⋯,R−1 **do**  3:    Sample subset $S^{r}\subseteq \left[N\right]$ of clients.  4:    $w_{i,0}^{t}=w^{r}$  5:    **for** each client $i\in S^{r}$ in parallel **do**  6:        **for** k=0,⋯,K−1 **do**  7:           Compute a cosine similarity-based classifier to optimize the  8:           class categories.  9:           Identify any newly added classes and adjust their weights10:           through the classifier.11:           Compute a local training estimate $g_{i,k}^{r}=\nabla F_{i}\left(w_{i,k}^{r},\xi_{i,k}^{r}\right)$ of $\nabla F_{i}\left(w_{i,k}^{r}\right)$.12:           Assign variable $L_{orth}=0$13:           **for** W in $\tilde{W}$ **do**14:               $L_{orth}=L_{orth}+\mathrm{DBT}\text{\_}\mathrm{Orth}\left(W\right)$15:           **end for**16:           Sum up the total loss $L_{total}$ by Equation (5)17:           $w_{i,k}^{r}=w_{i,k}^{r}-\eta_{l}\left(g_{i,k}^{r}+\lambda \cdot L_{total}\right)$.18:        **end for**19:        $\Delta_{i}^{r}=w_{i,K}^{r}-w^{r}$20:    **end for**21:    $\Delta^{r+1}=\frac{1}{S}\sum_{i\in S^{r}}\Delta_{i}^{r}$22:    $w^{r+1}=w^{r}+\eta_{g}\Delta^{r}$23:**end for**

### 3.2. Classifiers

In this study, we introduce classifier techniques into FedDyH with the aim of effectively addressing the challenges posed by data heterogeneity, particularly the issue of data category changes in dynamic data environments. In certain practical applications, the number of data categories is not fixed. As new categories are continuously introduced or old categories are removed, the distribution of data categories undergoes dynamic changes. For example, in insect classification research, deeper insights into ecological environments and genetic information may lead to new populations or subspecies being identified. In traditional classification methods, adding new categories typically requires retraining the entire model, involving the weights and parameters of all categories. This is highly resource-intensive and inefficient for handling complex dynamic data environments.

**Figure 2:**
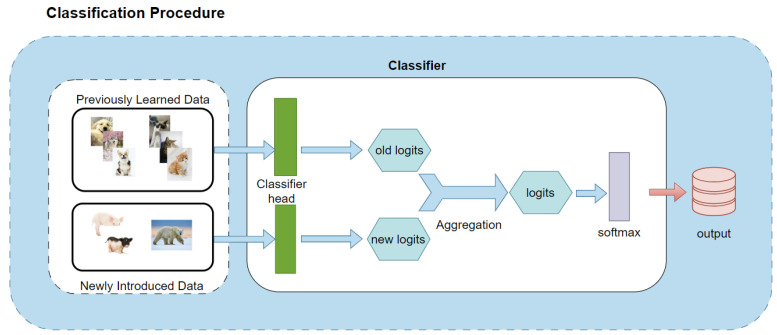
Working mechanism of cosine classifier.

To enhance the performance of the classifier in dynamic data environments, this study introduces a cosine similarity-based classification technique, which permits training only on newly added categories without modifying the weights of existing categories. This effectively reduces computational overhead and improves training efficiency. Additionally, we combine multi-agent learning and an adaptive attention mechanism, enabling the model to flexibly adjust its classification strategy based on the distribution of different categories in the feature space. This not only optimizes the model’s computational resource utilization but also significantly improves overall classification performance and accuracy. The specific working mechanism of the cosine classifier is shown in Figure 2.

The core of the cosine classifier is to perform classification by calculating the cosine similarity between the input features and the category prototype vectors. First, the input features and category prototype vectors are processed through L2 normalization to eliminate the impact of magnitude, allowing the classifier to focus on the directionality of the features. For a given input feature x and category weight w, their cosine similarity can be expressed as

(3) $$S\left(x,w\right)=-\frac{x\cdot w}{{∥x∥}_{2}{∥w∥}_{2}},$$

where x,w represents the dot product of the vectors and ${∥x∥}_{2}$ and ${∥w∥}_{2}$ represent the L2 norms of the feature and weight vectors, respectively, which can eliminate the influence of magnitude, allowing the classifier to focus solely on the direction of vectors. As a result, when the system needs to “add” or “remove” categories, it is only necessary to initialize or prune the corresponding prototype vectors and perform the same cosine similarity computation, without any need to alter the existing feature extraction or similarity measurement methods. Consequently, dynamically expanding or shrinking the number of categories will not disrupt the overall classification mechanism. Furthermore, to represent the diversity of categories, each category has multiple prototype vectors. Each prototype calculates its own similarity, and these similarities are weighted using adaptive attention coefficients α, ultimately yielding the score logits for that category. This process is completed by performing a weighted sum of the similarities for each prototype:

(4) $$logits_{c}=\sum_{m=1}^{p}\alpha_{c}^{m}\cdot S\left(x,w_{c}^{m}\right)$$

where *p* is the number of prototype vectors for each category and $\alpha_{c}^{m}$ is the attention coefficient for the m-th prototype vector of category *c*. By performing weighted aggregation of the similarity results from multiple prototype vectors within the same category, the model is able to capture the diverse representations of a category along different feature directions as well as to flexibly “add” or “remove” prototype vectors when the number of categories dynamically changes. Additionally, it can adjust the attention coefficients accordingly, ensuring that the evolving task requirements are met while maintaining a comprehensive representation and stable discrimination of each category. The attention coefficients α are calculated using the softmax function:

(5) $$\alpha_{c}^{m}=\frac{exp(\beta_{c}^{m})}{\sum_{m=1}^{p}exp\left(\beta_{c}^{m}\right)}$$

where $\beta_{c}^{m}$ is the learnable coefficient associated with the m-th agent vector. Another key feature of this classifier is its ability to dynamically adjust the number of categories. By adding or removing categories, the system can adaptively expand or shrink the category weights and the corresponding adaptive parameters α. When the number of categories changes, the classifier automatically initializes the weights based on the new number of categories and adjusts the number of prototypes for each category along with the corresponding α parameters. This enables the classifier to flexibly handle the challenges of changing category numbers while maintaining high discriminability and stability between categories, thereby ensuring accurate classification even in constantly changing data environments.

### 3.3. Regularization Strategies

After overcoming the challenges of dynamic data heterogeneity and client imbalance, we face the further problem of client models being prone to catastrophic forgetting in the face of new data and new tasks. Traditional federated learning assumes that data distributions are static and consistent, which makes it difficult for the global model to effectively adapt to the dynamic evolution of client tasks. Therefore, maintaining the stability of local knowledge and avoiding catastrophic forgetting under constantly changing data distributions represents a major challenge in model training.

To address this, we propose a new regularization strategy that combines orthogonality constraints and knowledge distillation techniques while computing the basic cross-entropy loss. Similar to the role of regulatory T cells in the immune system, this strategy balances the convergence of the global model with the adaptive adjustment of local specificity to ensure the stability and effectiveness of the model in dynamic data environments. Although this approach incurs some additional computational cost, the increase in computational overhead is relatively small compared to the performance improvement and can be considered negligible.

In the study of federated learning in dynamic heterogeneous environments, we introduce orthogonality constraints as a regularization method. This innovative approach was inspired by the successful application of orthogonality constraints in category-incremental learning by Q. Wei and W. Zhang et al. (2024) [27]. As shown in Equation (4), A is an $M\times C\times H^{\prime }W^{\prime }\times HW$ DBT matrix. A Toeplitz matrix is a matrix where the elements along each descending diagonal from left to right are the same, while a DBT matrix refers to a block Toeplitz matrix in which each block is itself a Toeplitz matrix. Here, $Toeplitz(w_{i})$ represents the Toeplitz matrix generated by the i-th row vector of convolution kernel w.

(6) $${\left[\begin{array}{cccc} T(w_{1}) & T(w_{2}) & \cdots & T(w_{n})\\ T(w_{2}) & T(w_{1}) & \cdots & T(w_{n-1})\\ \vdots & \vdots & \ddots & \vdots \\ T(w_{n}) & T(w_{n-1}) & \cdots & T(w_{1}) \end{array}\right]}_{\begin{array}{c} H^{\prime }W^{\prime }\\ \times \\ HW \end{array}}$$

Each “$Toeplitz(w_{i})$” block is a 2 × 2 Toeplitz matrix.

(7) $$T\left(w_{i}\right)=\left[\begin{array}{cc} w_{i} & w_{i+1}\\ w_{i+1} & w_{i} \end{array}\right]$$

First, we reformulate the 2D convolution kernel as a double-blocked Toeplitz (DBT) matrix. This matrix structure allows the convolution operation to be efficiently transformed into a matrix multiplication form, facilitating the application of orthogonality constraints. Next, we use the Frobenius norm to measure the difference between the product of the convolution kernel matrix and its transpose matrix on the one hand, and the identity matrix on the other. Based on this, we construct the orthogonality constraint loss function as shown in Equation (8). In the federated learning training process, we incorporate the orthogonality constraint loss as a regularization term into the total loss function of each client model:

(8) $$L_{orth}=||AA^{T}-I_{HW}{\left|\right|}_{F}$$

where $\left|\right|\cdot {\left|\right|}_{F}$ denotes the Frobenius norm, which is used to measure the similarity between models. This approach is effective in reducing overfitting, and improves the model’s performance on dynamically heterogeneous data.

To address the issues of fixed temperature parameters and sensitivity to feature scales in traditional knowledge distillation, we employ a dynamic adaptive distillation mechanism. The implementation of this mechanism involves three main steps: first, feature normalization preprocessing, where the logit outputs of the teacher and student models are Z-score normalized to eliminate the impact of feature scale differences on the soft target distribution; second, dynamic temperature adjustment, where a sample-level temperature coefficient is calculated based on the weighted standard deviation of the teacher model’s outputs, allowing difficult samples to automatically receive a higher degree of softening; and third, calculation of the distillation loss, which is computed using Kullback–Leibler (KL) divergence to measure the difference between the student model and the teacher model outputs, encouraging the student model to learn more generalizable knowledge from the teacher model and reducing the negative impact of client data heterogeneity on model training. Its distillation loss is

(9) $$L_{distill}=D_{KL}\left(\frac{P_{student}\left(y\right)}{T}|\frac{P_{teacher}\left(y\right)}{T}\right)\times \left(T^{2}\right),$$

where $P_{student}\left(y\right)$ and $P_{teacher}\left(y\right)$ represent the respective output probability distributions of the student and teacher models after temperature adjustment, $D_{KL}$ denotes the Kullback–Leibler divergence, which is used to measure the difference between the two probability distributions, and T is the temperature, which is used to adjust the smoothness of the output probability distribution. The squared temperature term in the formula helps to regulate the impact of the temperature on the distillation loss. This ensures that the student model learns the knowledge of the teacher model with an appropriate level of smoothness, enhancing the model’s robustness and generalization ability.

### 3.4. Genetic Algorithm

After addressing the issues of catastrophic forgetting and dynamic data heterogeneity, the system still needs to overcome the challenge of automatically optimizing the hyperparameters of the objective function. Given heterogeneous devices and dynamic data environments, the selection of the hyperparameters in the objective function has a significant impact on the model’s convergence speed and accuracy. In FedDyH, we propose a new regularization strategy in which the objective function consists of the cross-entropy loss, orthogonality loss, and distillation loss. The expression is as follows:

(10) $$L_{total}\left(\lambda_{distill},\lambda_{orth}\right)=L_{ce}+\lambda_{distill}\cdot L_{distill}+\lambda_{orth}\cdot L_{orth}$$

where $L_{ce}$ is the cross-entropy loss, $L_{distill}$ is the knowledge distillation loss, $L_{orth}$ is the orthogonality regularization loss, and $\lambda_{distill}$ and $\lambda_{orth}$ are the corresponding hyperparameters. By adjusting these two hyperparameters, it is possible to effectively control the relative importance of these two objectives during the training process, thereby optimizing the model’s performance.

To achieve the best performance of the objective function, we choose to use a genetic algorithm to optimize the hyperparameters of the distillation loss and orthogonality loss in the objective function. A genetic algorithm is a global optimization algorithm based on the principles of natural selection and genetics. Here, the GA simulates the process of biological evolution to gradually optimize the hyperparameter configuration through the mechanisms of genetic screening, crossover, and mutation. During the optimization process, the algorithm first randomly initializes a population in which each individual represents a set of hyperparameters. In each generation of the genetic algorithm, the fitness of each hyperparameter combination is calculated first. The fitness calculation formula is provided by Equation (11). Based on this calculation, high-quality individuals are selected for reproduction. The crossover operation involves two high-quality individuals exchanging features to produce new individuals. The mutation operation randomly alters some individual features to enhance population diversity. Through repeated iterations, the algorithm ultimately finds the optimal solution for hyperparameter optimization:

(11) $$Fitness\left(\lambda_{distill},\lambda_{orth}\right)=\frac{1}{1+L_{total}\left(\lambda_{distil}\cdot \lambda_{orth}\right)}$$

where $L_{total}\left(\lambda_{distill},\lambda_{orth}\right)$ is the value of the objective function. In this way, higher fitness indicates a smaller total loss, meaning that the hyperparameter combination is more optimal. In this formula, a smaller the total loss indicates a higher fitness value. Therefore, a hyperparameter combination with a higher fitness value corresponds to a lower total loss, implying better performance in optimizing the objective function. During the optimization process using the genetic algorithm, the fitness value is used to evaluate the quality of hyperparameter combinations in each generation, which in turn guides the selection, crossover, and mutation operations to achieve progressive optimization of the hyperparameter configurations.

To gain a deeper understanding of the performance and efficiency of our genetic algorithm, we conducted a detailed analysis of its key steps, assuming a population size of N and an optimization process spanning G generations. First, the algorithm needs to calculate the fitness values of all individuals, which is a crucial step in evaluating the quality of each individual. Because the fitness values need to be computed for N individuals and the computational complexity for each individual is typically O(1), the computational complexity of this stage is O(N). Next, the algorithm sorts individuals based on their fitness values and selects the top $\frac{N}{2}$ individuals as parents. The complexity of the sorting operation is primarily determined by the population size N, making the computational complexity of this stage O(NlogN). Subsequently, the algorithm generates offspring through crossover and mutation operations. These operations involve performing simple arithmetic calculations on half of the population ($\frac{N}{2}$ individuals), resulting in a computational complexity of O(N) for this stage. Overall, the computational complexity of each generation in the genetic algorithm is primarily influenced by three steps: fitness evaluation, sorting, and crossover/mutation, among which the sorting operation has the highest complexity at O(NlogN). Consequently, the overall computational complexity per generation is O(NlogN). If the algorithm runs for G generations, then the total computational complexity is O(GNlogN). In conclusion, although this approach introduces some additional computational cost, we believe that it is justified given the improvement in performance.

## 4. Experiments

In this section, we experimentally validate the feasibility and effectiveness of FedDyH. Experiments were primarily conducted on three public datasets and involved comparison with several advanced baseline methods. Specifically, the evaluation focused on the aspects of convergence speed and model accuracy.

### 4.1. Dataset and Model Setup

To validate the robustness and generalization ability of our method in different real-world scenarios, we selected three widely adopted and representative benchmark datasets in the federated learning (FL) field for evaluation: CIFAR-10, MNIST, and Fashion-MNIST. These datasets cover a range of learning tasks from simple to complex, allowing for comprehensive assessment of our method’s effectiveness.

Specifically, the CIFAR-10 [28] dataset contains 60,000 32 × 32 pixel color images divided into ten categories, with 6000 images per category. This dataset is widely used for image classification tasks and can effectively evaluate the model’s performance in handling complex visual features. The MNIST [29] dataset consists of 70,000 28 × 28 pixel grayscale images of handwritten digits, spanning ten categories from 0 to 9. It is a classic benchmark dataset in the field of image classification and is suitable for validating the performance of models on simple tasks. The Fashion-MNIST [30] dataset is an alternative version of MNIST consisting of 70,000 28 × 28 pixel grayscale images of fashion items such as T-shirts, pants, shoes, etc. It is divided into ten categories. Compared to MNIST, Fashion-MNIST presents a higher classification difficulty while retaining the same data format, making it suitable for validating the performance of models on more complex but still structured image classification tasks. Our experiments simulated real-world non-independent and identically distributed (Non-IID) data scenarios, allowing for an effective evaluation of the model’s performance under heterogeneous data distributions. Conducting experiments on three datasets, each with different characteristics and challenges, allows us to comprehensively assess the effectiveness and robustness of the proposed method across various federated learning scenarios.

In terms of model settings, we selected appropriate network architectures based on the characteristics of each dataset. The specific parameter implementations are shown in Table 2, Table 3 and Table 4.

### 4.2. Implementation Details

Our experiments were conducted in the following hardware and software environment: the hardware environment consisted of a 16 vCPU Intel(R) Xeon(R) Platinum 8352V CPU @ 2.10 GHz and an NVIDIA RTX 4090 GPU, while the software environment was Ubuntu 20.04. The project was deployed based on PyTorch 1.11.0, Python 3.8, and CUDA 11.3. In the experiments, we used the SGD optimizer with a weight decay of 1e-5, momentum of 0.9, and training batch size of 32. The default learning rate was set to 0.01, and the number of clients was 50. For the CIFAR-10 dataset, the learning rate was adjusted to 0.1, with a single local epoch and 100 global communication rounds. To ensure fairness among the clients, all clients initially contained the same number of fixed data samples.

To validate the effectiveness of our method, we selected several state-of-the-art algorithms that perform well in heterogeneous federated learning scenarios for comparison. These methods included FedDecorr [31] and FedSAM [32], which are local optimization methods proposed at recent top conferences such as ICLR 2023 and ICML 2022 and have demonstrated significant performance improvements in static heterogeneity scenarios. FedExp [33] is an advanced server-based optimization method, also published at the top conference ICML 2023, which effectively improves the convergence of the global model. FedProx [12] introduces a proximal term in the local objective function to minimize client drift. FedAvg, as the most basic baseline method in federated learning, is used for comparative validation of the superiority of our method. The above experimental setup and selection of comparison methods allows for a comprehensive evaluation of the effectiveness and robustness of the proposed method in heterogeneous federated learning scenarios.

### 4.3. Experiment Analysis

#### 4.3.1. Ablation Experiments

To validate the effectiveness of each innovative component in our method, we performed cross-validation on the CIFAR-10 dataset. Table 5 demonstrates the evaluation of the four innovative components, i.e., classifiers, orthogonality constraints, knowledge distillation techniques, and genetic algorithm, with a degree of heterogeneity of 0.5 and client selection rate of 0.5. The experimental results show that the accuracy continues to improve as the different techniques are gradually incorporated. Initially, without any additional techniques, the accuracy is the lowest at only 47.13%. After introducing the classifier, the accuracy improves, indicating that the classification technique based on cosine similarity effectively preserves local features in dynamic data environments while avoiding the interference caused by frequent modifications to existing category weights. Next, when separately evaluating the classifier combined with orthogonality constraints and the classifier combined with knowledge distillation, the results are 51.73% and 51.89%, respectively. These results show little difference in accuracy, with both versions improving on the use of the classifier alone. Thus, benefits arise from the use of orthogonality constraints as a regularization method, which effectively reduces overfitting, as well as from knowledge distillation, which enables sharing of core knowledge among clients in order to mitigate the forgetting problem caused by shifts in the data distribution. When combining all three components, the model performance improved significantly. This indicates that the proposed series of strategies effectively mitigates the problems of imbalanced data and forgetting. Finally, combining all three methods and further introducing the genetic algorithm for optimization achieved the best results, confirming that the synergistic effect of these techniques significantly enhances the model’s performance. These results validate the significant gains provided by the genetic algorithm by leveraging gene selection, crossover, and mutation processes to automatically optimize the hyperparameter configuration.

#### 4.3.2. Comparison with Advanced Methods Under Different Heterogeneity Levels

**Figure 3:**
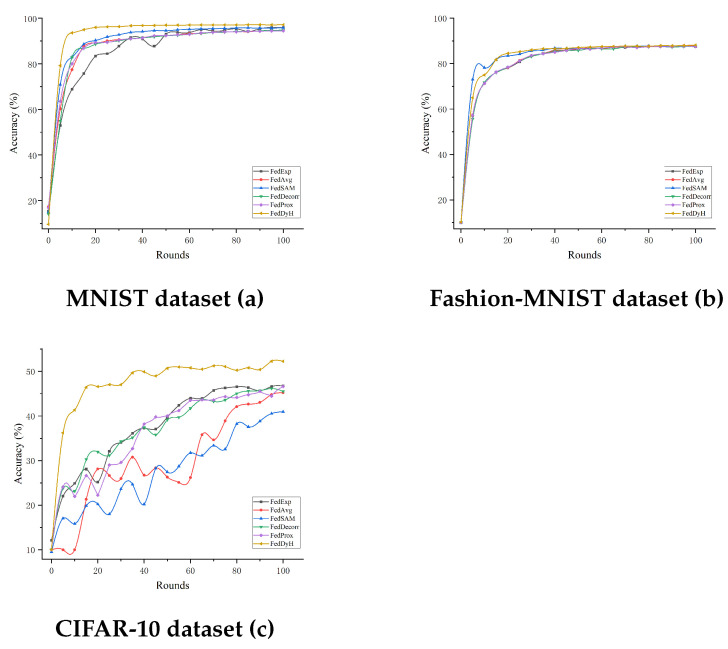
Performance comparison on the three datasets for a heterogeneity degree of 0.5.

**Figure 4:**
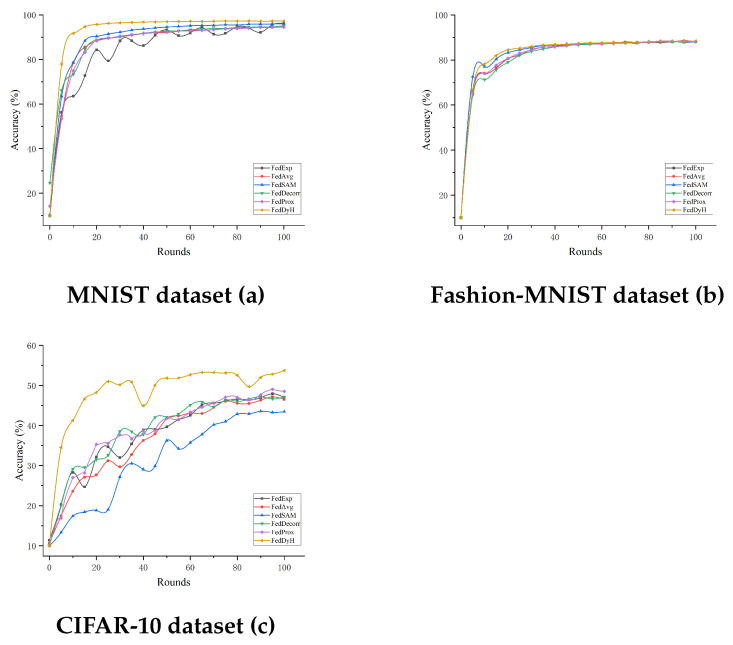
Performance comparison on the three datasets for a heterogeneity degree of 0.1.

The aim of this study is to evaluate the efficacy of the proposed FedDyH method for client training in a dynamic heterogeneous environment. Considering the time-varying nature of data distributions in real-world applications, which may lead to the dynamic evolution of heterogeneity, we systematically set two significantly different heterogeneity coefficients of 0.1 and 0.5 in our experimental design in order to construct non-IID scenarios with varying levels of intensity. The experimental results are shown in Figure 3 and Figure 4 and Table 6, where it can be clearly seen that FedDyH shows a significant advantage in dealing with high heterogeneity on different datasets. FedDyH consistently demonstrates faster convergence and higher final accuracy on the MNIST, FMNIST, and CIFAR-10 datasets when the degree of heterogeneity increases to 0.5. This advantage is particularly pronounced on the CIFAR-10 dataset, where the feature space is more complex. Although other methods such as FedExp and FedAvg perform reasonably well in the early rounds, their generalization ability becomes limited as new categories are gradually introduced and heterogeneity increases, leading to a slowdown in convergence speed and significantly lower final accuracy than that FedDyH. In contrast, FedDyH demonstrates excellent adaptability to heterogeneity thanks to its dynamic adjustment mechanism, maintaining high accuracy even in more complex scenarios.

#### 4.3.3. Comparison with Advanced Methods Under Different Client Selection Rates

**Figure 5:**
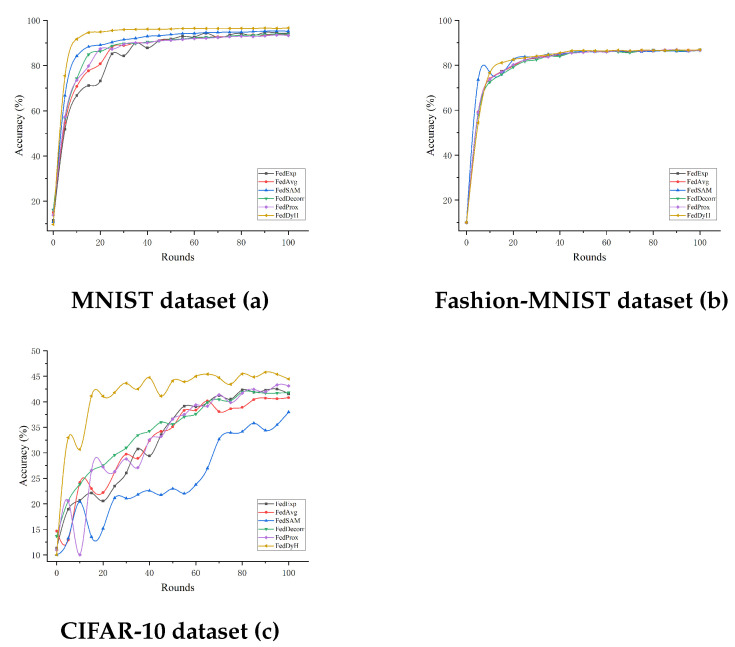
Performance comparison with a client selection rate of 0.2 on the three datasets.

For the experiments with a heterogeneity degree of 0.5, we used client selection rates of 0.2 and 0.5 for comparison in order to simulate different client selection scenarios. Having already validated the performance of our proposed method with a client selection rate of 0.5 in the previous experiments, we conducted experiments with a client selection rate of 0.2 to further validate its effectiveness. As shown in Figure 5 and Table 7, it is clear that FedDyH consistently demonstrates excellent performance with client selection rates of both 0.2 and 0.5, far outperforming the other methods. When the client selection rate is 0.2, the accuracy of all methods is generally lower, but FedDyH still manages to maintain a high level of accuracy. This is particularly evident on the CIFAR-10 dataset, where FedDyH completes the training process with a clear and significant advantage. In contrast, methods such as FedProx and FedAvg exhibit slower convergence and slightly lower final accuracy, indicating that FedDyH’s dynamic optimization strategy effectively mitigates the negative impact caused by the continuous introduction of new categories. Although FedSAM and FedDecorrr show some improvement in the later stages of training, they still fail to surpass FedDyH, reflecting their lower adaptability when fewer clients are selected. When the client selection rate is increased to 0.5, we observe a general improvement in the performance of all methods, as the involvement of more clients helps to accelerate the learning process. Nonetheless, the advantage of FedDyH remains highly evident. Compared to the client selection rate of 0.2, the accuracy of FedDyH further improves under the 0.5 selection rate condition, with a significant increase in convergence speed. This is especially noticeable on the CIFAR-10 dataset, where its performance stands out remarkably. However, compared to the other two datasets, it is evident that the curve for FedDyH exhibits significant fluctuations between the 30th and 50th rounds on the CIFAR-10 dataset. This is related to its dynamic client weight adjustment strategy, which causes the loss values of the local models to fluctuate more during more complex tasks, leading to unstable weight allocations.

#### 4.3.4. Impact of Local Training Rounds

**Figure 6:**
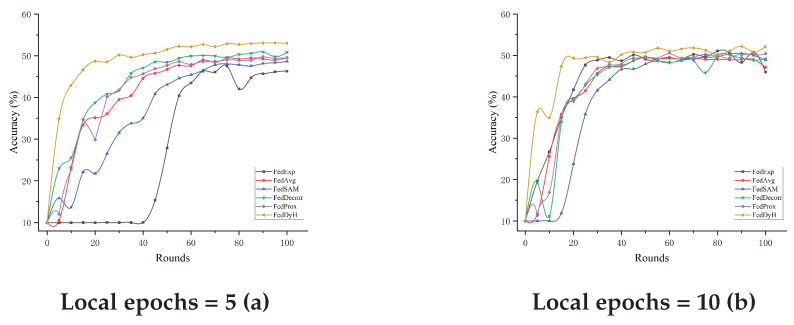
Performance comparison of the different methods with varying local training epochs.

We conducted experiments on the CIFAR-10 dataset to assess the impact of local training rounds with the degree of heterogeneity and client selection rate both to 0.5. The experimental results are shown in Table 8 and Figure 6. FedDyH exhibits significant advantages with different numbers of local training rounds, especially when there are fewer training rounds. Thanks to its regularization method combining orthogonality constraints with knowledge distillation, FedDyH can effectively improve the robustness of the model and prevent overfitting. With a single round of local training, FedDyH leads the other methods with an accuracy of 53.71%, and its dynamic tuning mechanism fully utilizes the limited data to rapidly improve the accuracy of the model. In contrast, methods such as FedExp and FedAvg perform relatively poorly in the early training stages. Although FedDyH’s accuracy decreases slightly when increasing the number of local training rounds, it still maintains a significant advantage. Through experimental analysis, we found that FedDyH’s dynamic tuning mechanism may lead to overtuning of the model with longer local training rounds, and may fail to sufficiently suppress client-side overfitting. However, this phenomenon did not weaken the overall performance of FedDyH; on the contrary, the intervention using multiple strategies still led to significant performance improvements in most cases.

#### 4.3.5. Hyperparametric Sensitivity Analysis

To verify the effectiveness of our hyperparameter optimization method, we conducted a sensitivity analysis experiment. As shown in Table 9, we selected three manually set empirical hyperparameter configurations and compared them with our proposed GA-based adaptive optimization method for validation. The experimental results indicate that GA optimization yields only a minor improvement of +0.05% compared to the best manually set configuration on low-complexity tasks, suggesting that the hyperparameter tuning space is relatively limited for such tasks. However, on the more complex Fashion-MNIST and CIFAR-10 datasets, GA optimization achieves significant performance gains of +1.08% and +1.18%, respectively, validating the advantage of dynamic optimization methods in high-dimensional feature regulation. Optimizing the hyperparameters using the GA can effectively avoid the local optimum trap in manually set configurations. Although this approach introduces some additional computational cost, we believe that it is worthwhile in light of the resulting performance improvements.

## 5. Discussion

Our experimental results show that FedDyH demonstrates a significant improvement in accuracy during global model training, particularly when handling heterogeneous data and scenarios with limited local training rounds. Compared to the baseline FedAvg method, FedDyH incurs approximately 40% higher computational cost, while its communication cost remains unchanged. Although this additional computational overhead imposes higher demands on both client and server processing capabilities, the substantial performance gains justify this investment. Thus, despite the increase in computational cost, the enhanced performance of our proposed FedDyH method clearly showcases its advantages in real-world applications. By integrating multiple innovative strategies, FedDyH not only outperforms traditional federated learning methods across various datasets but also effectively avoids the overfitting problem, further enhancing its stability and adaptability. Although FedDyH demonstrated strong performance in our experiments, there is still room for improvement. For example, the efficiency and accuracy of hyperparameter optimization need further improvement, and the issue of catastrophic forgetting may become more pronounced in environments with larger-scale and more rapidly changing data. Furthermore, adaptability to client heterogeneity still requires additional enhancement.

## 6. Conclusions

This paper introduces the FedDyH framework, which integrates multiple optimization strategies to collectively enhance model performance. Through the synergistic effects of integrating our proposed classifier, orthogonality constraints, knowledge distillation regularization, and genetic algorithm, FedDyH effectively addresses challenges such as dynamic data heterogeneity and catastrophic forgetting to ensure the resulting model’s adaptability in evolving environments. Our experimental results demonstrate that FedDyH excels in multiple aspects, highlighting its broad potential and advantages in real-world applications.

In future work, we plan to further explore the application of FedDyH in more complex scenarios, including handling datasets of varying scales and environments with higher degrees of heterogeneity. As the number of clients increases, scalability will become a critical issue. To address this, we plan to explore how parallel computing, distributed strategies, and dynamic client selection can be leveraged to handle the challenges of large-scale client scenarios. Furthermore, from the perspective of real-world applicability, FedDyH may encounter several challenges during actual deployment; examples include efficiently implementing a dynamic adjustment mechanism in environments with limited or unstable network bandwidth as well as properly handling significant differences in client hardware resources. As a next step, we will conduct an in-depth investigation and exploration of these practical issues. Additionally, we will explore integrating other advanced optimization techniques into FedDyH as a way to further enhance its efficiency and generalization ability in federated learning. Through continuous efforts and optimization, we believe that federated learning algorithms will play an increasingly important role across various domains.

## Figures and Tables

**Table 1:** Comparison of FedDyH with other federated learning methods.

Method	Adaptability to Dynamic Heterogeneous Scenarios	Optimization Strategy	Catastrophic Forgetting Mitigation	Hyperparameter Optimization	Data Heterogeneity Handling	Model Generalization Capability
**FedAvg**	Weak (assumes static data)	Traditional gradient averaging	None	None	Weak (suitable for IID data)	Moderate (struggles with heterogeneous data)
**FedProx**	Moderate (partially adapts to heterogeneous data)	Traditional gradient optimization + proximal term	None	None	Moderate (constrains local drift)	Moderate (limited by regularization strength)
**SCAFFOLD**	Moderate (partially adapts to heterogeneous data)	Control variate adjustment (reduces local drift)	Weak (not explicitly considered)	None	Moderate (control variate adjustment)	Moderate (depends on data quality)
**FedSAM**	Moderate (enhanced robustness)	SAM optimization (improves smoothness)	None	None	Moderate (adjusts local models)	Strong (improves model robustness)
**FedDercorr**	Moderate (reduces data correlation)	Decorrelation-based optimization	None	None	Strong (decorrelation regularization)	Strong (effective for heterogeneous data)
**Our**	Strong (supports dynamic data changes)	Cosine classifier + orthogonality constraint + knowledge distillation + genetic algorithm	Strong (knowledge distillation reduces forgetting)	Genetic algorithm for adaptive optimization	Strong (orthogonality constraint for regularization)	Strong (dynamic model adjustment)

**Table 2:** Applied MNIST CNN model architecture.

Layer	Kernel Size/Pool Size/Rate	Output Shape	Param #
Conv2D	(5, 5)	(64, 24, 24)	1664
Conv2D	(5, 5)	(64, 20, 20)	102,464
Dropout	0.25	(64, 20, 20)	0
Flatten	–	(25,600)	0
Dense	–	(128)	3,276,928
Dropout	0.5	(128)	0
CosineClassifier	–	(10)	1290

**Table 3:** Applied FashionMNIST CNN model architecture.

Layer	Kernel Size/Pool Size/Rate	Output Shape	Param #
Conv2D	(3, 3)	(128, 28, 28)	1280
Conv2D	(3, 3)	(128, 28, 28)	147,584
Conv2D	(3, 3)	(128, 28, 28)	147,584
MaxPooling	(2, 2)	(128, 14, 14)	0
Dropout	0.25	(128, 14, 14)	0
Conv2D	(3, 3)	(256, 14, 14)	295,168
Conv2D	(3, 3)	(256, 14, 14)	590,080
Conv2D	(3, 3)	(256, 14, 14)	590,080
MaxPooling	(2, 2)	(256, 7, 7)	0
Dropout	0.25	(256, 7, 7)	0
Conv2D	(3, 3)	(512, 5, 5)	1,180,160
Conv2D	(3, 3)	(256, 3, 3)	1,179,904
Conv2D	(3, 3)	(128, 1, 1)	295,040
AvgPooling	(1, 1)	(128, 1, 1)	0
Flatten	–	(128)	0
CosineClassifier	–	(10)	1290

**Table 4:** Applied CIFAR-10 ResNet model architecture.

Layer	Kernel Size/Pool Size/Rate	Output Shape	Param #
Conv2D (conv1)	(7, 7), stride = 2	(64, 16, 16)	9472
BatchNorm2d (bn1)	–	(64, 16, 16)	128
MaxPool2d	(3, 3), stride = 2	(64, 8, 8)	0
ResBlock1 (x2)	[(3, 3),(3, 3)] × 2	(64, 8, 8)	148,224
ResBlock2 (x2)	[(3, 3),(3, 3)] × 2	(128, 4, 4)	526,336
ResBlock3 (x2)	[(3, 3),(3, 3)] × 2	(256, 2, 2)	2,099,200
ResBlock4 (x2)	[(3, 3),(3, 3)] × 2	(512, 1, 1)	8,392,960
AvgPool2d	Adaptive(1, 1)	(512, 1, 1)	0
Flatten	–	(512)	0
CosineClassifier	–	(10)	5130

**Table 5:** Quantitative ablation study with a heterogeneity degree of 0.5 and client selection rate of 0.5 on the CIFAR-10 dataset.

Classifier	Orth	Distill	GA	ACC (%)
				47.13
✓				51.48
✓	✓			51.73
✓		✓		51.89
✓	✓	✓		53.05
✓	✓	✓	✓	53.71

**Table 6:** Comparison of model accuracy on the three datasets for heterogeneity degrees of 0.1 and 0.5.

Method	MNIST		Fashion-MNIST		CIFAR-10	
	**0.1**	**0.5**	**0.1**	**0.5**	**0.1**	**0.5**
FedAvg	93.49 ± 0.15	94.14 ± 0.21	88.09 ± 0.14	88.43 ± 0.18	45.42 ± 0.13	47.13 ± 0.33
FedProx	94.56 ± 0.24	94.49 ± 0.24	87.74 ± 0.12	88.56 ± 0.16	46.62 ± 0.14	49.06 ± 0.17
FedExp	95.88 ± 0.21	96.19 ± 0.14	87.92 ± 0.25	88.30 ± 0.16	47.39 ± 0.34	48.34 ± 0.29
FedSAM	96.11 ± 0.33	96.02 ± 0.27	88.26 ± 0.28	88.14 ± 0.16	44.27 ± 0.27	44.27 ± 0.27
FedDcorr	94.64 ± 0.29	95.24 ± 0.11	87.69 ± 0.12	88.15 ± 0.17	46.67 ± 0.17	47.89 ± 0.12
**FedDyH**	**97.23 ± 0.14**	**97.42 ± 0.16**	**88.24 ± 0.21**	**89.13 ± 0.18**	**52.46 ± 0.17**	**53.71 ± 0.15**

**Table 7:** Accuracy comparison for client selection rates of 0.2 and 0.5 on the three datasets.

Method	MNIST		Fashion-MNIST		CIFAR-10	
	**0.2**	**0.5**	**0.2**	**0.5**	**0.2**	**0.5**
FedAvg	93.26 ± 0.14	94.14 ± 0.21	86.89 ± 0.23	88.43 ± 0.18	41.40 ± 0.19	47.13 ± 0.33
FedProx	93.50 ± 0.15	94.49 ± 0.24	86.97 ± 0.14	88.56 ± 0.16	44.02 ± 0.17	49.06 ± 0.17
FedExp	94.28 ± 0.25	96.19 ± 0.14	86.84 ± 0.24	88.30 ± 0.16	43.12 ± 0.17	48.34 ± 0.29
FedSAM	95.26 ± 0.13	96.02 ± 0.27	86.72 ± 0.14	88.14 ± 0.16	37.86 ± 0.14	44.27 ± 0.27
FedDcorr	93.96 ± 0.29	95.24 ± 0.11	86.84 ± 0.14	88.15 ± 0.17	42.90 ± 0.16	47.89 ± 0.12
**FedDyH**	**96.59 ± 0.19**	**97.42 ± 0.16**	**87.02 ± 0.23**	**89.13 ± 0.18**	**45.83 ± 0.24**	**53.71 ± 0.15**

**Table 8:** Accuracy with 1, 5, and 10 local rounds.

Method	1	5	10
FedAvg	47.13 ± 0.33	49.53 ± 0.14	50.12 ± 0.26
FedProx	49.06 ± 0.17	49.99 ± 0.18	50.53 ± 0.15
FedExp	48.34 ± 0.29	47.59 ± 0.21	51.35 ± 0.25
FedSAM	44.27 ± 0.27	49.64 ± 0.17	50.52 ± 0.21
FedDcorr	47.89 ± 0.12	50.87 ± 0.13	50.14 ± 0.18
**FedDyH**	**53.71 ± 0.15**	**53.19 ± 0.15**	**52.15 ± 0.14**

**Table 9:** Performance comparison between GA-optimized parameters and manually set parameters.

Method	MNIST	Fashion-MNIST	CIFAR-10
$\lambda_{orth}$ = 0.5 $\lambda_{distill}$ = 0.5	97.23%	87.72%	50.88%
$\lambda_{orth}$ = 0.2 $\lambda_{distill}$ = 0.8	97.28%	87.98%	52.06%
$\lambda_{orth}$ = 0.1 $\lambda_{distill}$ = 0.9	97.27%	88.05%	52.53%
**GA Optimisation**	**97.32%**	**89.13%**	**53.71%**

## Data Availability

The research data can be obtained from the corresponding authors upon reasonable request. The datasets are available online.
